# The “One Mind, Two Aspects” Model of the Self: The Self Model and Self-Cultivation Theory of Chinese Buddhism

**DOI:** 10.3389/fpsyg.2021.652465

**Published:** 2021-04-28

**Authors:** Kai Wang

**Affiliations:** ^1^Department of Philosophy, Nanjing University, Nanjing, China; ^2^Department of Oriental Languages and Cultures, Ghent University, Ghent, Belgium

**Keywords:** self model, moral self, one mind, two aspects, Chinese Buddhist culture

## Abstract

Constructing a self model with universal cultural adaptability is a common concern of cultural psychologists. These models can be divided into two types: one is the self model based on Western culture, represented by the self theory of Marsh, Cooley, Fitts, etc.; the other is the non-self model based on Eastern culture, represented by the Mandela model of Hwang Kwang Kuo and the Taiji model of Zhen Dong Wang. However, these models do not fully explain the self structure and development of Chinese people in the context of Chinese Buddhist culture. Based on the self theory of Chinese Buddhism and inspired by the famous Buddhist work *Awakening of Faith in the Mahāyāna*, this article constructs the “one mind, two aspects” self model. This model not only can properly represent the self structure of Chinese people in the context of Chinese Buddhism but also can explain the self-cultivation process and the realm of practice of Chinese Buddhist believers and thus has satisfactory cultural validity.

## Introduction

Constructing a self model has been an important long-standing issue for psychologists (Zhan and Yue, 2002). Psychologists initially tried to construct a universal self model that can generally explain people's mental structure based on a single culture (William, 1890; Cooley, 1902; Mead, 1934). In recent decades, with the rise of cross-cultural psychology, an increasing number of psychologists have found it difficult to have a self model based on a single culture that can be universally applied to all cultural situations (Rogers, 1951). Therefore, they began to explore diverse self models based on different cultural contexts. These diverse models can be divided into the self model and the non-self model. The self model is based on Western egoism, which is a process of using the principle of hedonism to pursue desire. The non-self model is based on Eastern Tibetan Buddhism, Chinese Confucianism, and Taoist culture, and emphasizes the elimination of one's desires in order to obtain maximum happiness. Both models have limitations: neither model talks about the “one mind, two aspects” self model created by Chinese Buddhism, which has greatly influenced the self theory in traditional Chinese culture and can compensate for the shortcomings of the two self model theories noted above.

## Self Models and Their Limitations

The exploration of self has always been an important issue throughout the history of philosophy. Early philosophers represented by Socrates aimed to explore the nature of the self. In modern times, influenced by the development of natural science, Descartes and other philosophers began to explore the structure and function of the self from the perspective of epistemology. In the 19th century, psychology, as a science, was independent from philosophy and inherited the discussion of the concept of “self” in philosophy. Influenced by modern science, the construction of a set of self model with universal applicability has become the focus of psychological self-research. With the continuous enrichment of self-study paradigm, the construction of self model has gradually formed two research directions: scientism self-study and humanism self-study (Gergen, 2001; Giorgi, 2005).

### Researches on the Object Self Models From the Perspective of Individualism

Since Hume and Descartes, philosophers have generally paid attention to such a problem: the existence of any human experience requires the existence of a self, the self is the premise of the existence of human experience, and the self, as a transcendental concept, cannot be understood through experience. This gives rise to an epistemological paradox about the self, which has become the focus of philosophical self-study (Shear and Gallagher, 2013). In contrast, scientism self study puts the empirical science as its methodology, the individualism as its theoretical foundation, pays attention to be different from an philosophical abstract analysis of self concept. It emphasizes the objective attribute of the self, shifts the research focus from the essence of the self to the structure and function of the self, devotes itself to analyzing the characteristics of people's self-concept and its formation and development process, pursues the consistency and integration of the self, and builds the self into a constant entity. In reality, it emphasizes the uniqueness of the individual self.

William James was the pioneer of scientism self study. In 1892, William James first divided the “self” into two aspects: subject and object, constructed the subject–object model of self. In this model, the “Me,” as the object, represents the individual's own experience, whereas the “I,” as the subject, represents the individual's perception of their own activities and life experience (William, 1892). In James' opinion, the object self Me is concrete and operable, which is based on experience, and ought to be the object of psychological scientific research. However, the subject self I comes from the research tradition of philosophical concepts in essence and is speculative, so it should not be concerned by psychology (Oosterwegel et al., 1993). James' emphasis on the object self greatly influenced the subsequent study of the self in scientific psychology. More and more psychologists regarded the self as an entity formed by the collection of experience and began to study the structure and function of the object self in order to construct more specific models of object self. Existing researches mainly include the following two categories.

One focuses on the structural changes and characteristics of the object self in different situations and different time stages. James argues that the object self varies with the context in which experience is acquired. Therefore, the structure of the object self will change with the environment and time of the individual (William, 1890). On the basis of this view, Mead focuses on the analysis of the influence of time on the formation of individual self-concept. He believes that although individuals always live in the “now” in reality, they can perceive the “past” and “future” through the present experience and form the concept of past self and future self (Mead, 1934). In 1988, Neiser made it clear that the individual's self-concept is malleable in time, that is, from the perspective of time, the self is a so-called “temporally extended self,” which can be divided into past self, present self, and future self in structure (Neisser, 1988). Thus, an object self model with temporal characteristics is formed.

The other focuses on the structural characteristics of the object self at different levels. This type of object self research focuses on the areas of body psychology and moral psychology. In physical psychology research, the earliest and most influential self model is the psychological model of physical activity constructed by Sonstroem. In this model, Sonstroem emphasized that perceptions of physical ability and self-acceptance form the two basic elements for establishing a sense of self-worth. The perception of physical ability refers to an individual's assessment of their own physical ability; self-acceptance is the degree to which an individual likes and cares about himself. When the two are actively promoted, the sense of self-worth increases, and the individual's sense of self-identity also increases. The improvement of these two elements depends on the improvement of self-efficacy brought about by physical exercise (Sonstroem and Morgan, 1989). Fox and Corbin further refined the sense of self-worth into four dimensions: perception of athletic ability, physical condition, physical attraction, and strength (Fox and Corbin, 1989). Marsh further divided the self into nine dimensions: strength, thinness, mobility, endurance, athletic ability, coordination, health, appearance, and flexibility. The weights of these nine dimensions in self-identity were also discussed (Marsh and Redmayne, 1994). All studies relate to the physical reinforcement of self-worth. In the study of moral psychology, Fitts developed the Tennessee self-concept scale. The scale divides the self concept into five dimensions: physical, moral–ethical, personal, family, and social. Each dimension sets questions from the three angles of self-identity, self-satisfaction, and self-behavior, so as to evaluate the formation of individuals' self concept (Fitts, 1965). Marsh added the dimension of moral self into the Shavelson self model and took religious belief and honesty as the basic elements of moral self construction (Marsh, 1992). Walker used cluster analysis to further clarify the eight characteristics of the mature moral self: principle, idealization, loyalty, perfection, care, trust, fairness, and self-confidence (Walker, 2004). Since then, moral self has become an integral part of the self model.

Although the study of the object self greatly promoted the people's understanding of the experience of self, it is undeniable that there are still certain theoretical limitations; as summarized by Yung, object self study and model on the basis of the formation of a variety of self, more or less derived from the protestant, the influence of American individualism self concept will be the formation of individual self concept based on the following three theoretical principles: the first is individuals can sense the formation and changes of self concept; the second is the psychological entity composed of the perception, desire, demand, and mental function of the biological individual; thirdly, the self-concept of biological individuals is a kind of psychological function that should be recognized (Shiah, 2016). The purpose of understanding oneself is to stimulate self-potential and realize self-satisfaction (Yang and Lu, 2009). So, in general, the object of the self study in America under the influence of cognitive psychology of the individual self concept is a paradigm in the study, its theory essence is to the individual self as substantive exists, the analysis of its own and development factors to affect the change of the analysis is in order to facilitate the individual more in-depth understanding of the self, in order to promote the individual happiness. Such emphasis on the individual and the praise of individualism obviously ignores those “those mental products which are created by a community of human life and are, therefore, inexplicable in terms merely of individual consciousness” (Wundt, 1916) and cannot explain the self-cognition formed when individuals gather into collectives. Social psychologists have noticed this limitation and have tried to construct a new self theory model from the perspective of collectivism in order to improve the study of psychological self.

### Researches on the Object Self Models From the Perspective of Collectivism

Although Wundt discovered as early as 1912 the limitations of individualism self model and tried to let the psychologists focus on the collective self in the field of social psychology, the shadow of the American individualism in cognitive psychology for a long time occupies the mainstream position in the field of psychological research, so the process of the construction of the collective self had its twists and turns (Hogg and Williams, 2000).

As early as the beginning of the 20th century, Lebon, Tarde, and others in the field of social psychology focused their research on the crowd (Tarde, 1901; LeBon, 1908). McDougall also points out that in the interaction between individuals, there is a kind of “group mind,” which has a reality and existence qualitatively different from the isolated individuals who compose the group (McDougall, 1921). Mead examined the social experience arising from the interaction between individuals and a variety of social situations, and its effects on the formation of individual self concept. Mead insisted that this kind of interaction will cause the change of the self structure of individuals and will affect the individual's perception of himself/herself, that is to say, the individual's self concept in addition from the individual's own experience, some from social experience. Accordingly, he believes that the object self model as the collection of individual experience should contain two parts: individual self and social self (Mead, 1925, 1934). Moscovici et al. subsequently conducted the latest research on the emergence of social representation in social interaction and further confirmed that human interaction in the crowd has emergent characteristics that influence others (Moscovici, 1984). All these theories contribute to the construction of the collective self model, but they have been criticized by Allport et al. According to Allport (1962), the collective self is also a psychological entity constructed by individual experience, which does not get rid of individualism. Allport also points out that “There is no psychology of groups which is not essentially and entirely a psychology of individuals” (Graumann, 1986). Since then, the study of the collective self has returned to the study of the individualistic self. By the 1920s, there was no longer a relevant study of the collective self in the social psychology research (Hogg and Williams, 2000).

In the 1970s, psychologists realized that treating the collective mind solely as a collection of individual minds could not adequately explain large-scale group phenomena such as intergroup conflict, social protest, and social change (Cartwright, 1979). So, through the reflection of previous researches on the collective self, European psychologists put forward the theory of social identity and began to construct the self from the perspective of group (collective/crowd) again. The so-called social identity, according to Tajfel, refers to an individual's knowledge that he belongs to some social group, as well as the emotional and value significance of the members of this group to him. This kind of understanding will form the sense of belonging of the individual self, so as to obtain a certain satisfaction. When a group is compared with other groups, members of the group will make a self-evaluation that is beneficial to their own group according to the differences between groups, so as to enhance their positive uniqueness and positive social identity, thus forming a collective self-concept based on the group (Tajfel, 1972, 1974). Brewer further pointed out that the collective self is the highest level of conceptualization and abstraction of individual self-cognition, which can overcome the deficiency of the object self model formed based on individual experience in explaining group phenomenon (Brewer, 1988).

The construction of collective self overcomes the limitation that the object self model based on individualism relies too much on individual experience, but because its theoretical background is American individualism and European humanism, it has produced new limitations in the interpretation of self-concept in different cultures. For example, collective self aims at pursuing consistency and integration. When the individual is in a group different from other groups, it advocates to maintain the consistency and stability of the self-group through self-evaluation, so as to obtain psychological satisfaction. This obviously does not explain the self-concept in Eastern cultures, which are also collectivist, where the self is encouraged to constantly change as the environment changes (Yang and Lu, 2009). For another example, the ultimate goal of collective self theory is to enhance the sense of belonging, satisfaction, and happiness of individuals, which is also incompatible with the Eastern culture, which emphasizes that self-sacrifice should be decisive in the collective (Feng, 1933).

This defect has attracted the attention of cultural psychologists in the past decade. They try to build more universal self models from different cultural backgrounds. In 1989, Triandis compared the probabilities of three different types of self in different social environments to demonstrate the influence of different cultures on the formation of the self concept. These three kinds of selves are the private self, which involves a person's cognition of his own characteristics, state, or behavior; the public self, concerning the general human self-cognition; and the collective self, concerning some collective self views (Triandis, 1989). This kind of self division based on different cultures was supported by Brewer and Gardner (Brewer and Gardner, 1996). Another independent study conducted by Markus and Kitayama also suggested that people in different cultures have surprisingly different psychological structures in their understanding of the self and the other. Most of the self formed under the influence of Western culture is independent, which is shown as a bounded, self-sufficient, and independent entity, emphasizing the separation of individual and social backgrounds. In contrast, the self formed under the influence of East Asian culture is interdependent and pays more attention to social relations and the connection with the environment (Markus and Kitayama, 1991).

These theories have made remarkable contributions to describing the establishment and development of the self, but they lack philosophical analysis of the interaction between culture and the self (Talhelm et al., 2014; Zhu and Ng, 2017). Because of this, they cannot fully explain the self concept, self structure, or self development in the background of Oriental, and particularly Chinese, culture with a special emphasis on the absolute abandonment of the individual self (Wang et al., 2019).

## Non-self Models and Their Limitations

In recent years, an increasing number of psychologists have begun to explore the self model based on Oriental culture because of the defects of Western self theory in explaining heterogeneous culture. These models are usually called “non-self” to show the characteristics of self deconstruction in Eastern culture. Although these models are highly diverse, they cannot explain the self model in Chinese Buddhism. The following provides analysis of two representative models to prove this point.

### The Mandala Model of the Self

Because psychology has long explained some psychological mechanisms through the theory of Tibetan Buddhism, the self models based on the construction of Tibetan Buddhist culture have been the subject of wide interest within psychological circles. Among these models, the most representative is the mandala model of the self.

The mandala is a symbol originating from Indian religion. Its basic form is a circle, which is usually used to refer to the universe. This symbol was later adopted by Buddhism and is very common in Tibetan Buddhism. In 1963, Jung used the mandala symbol to describe the self and defined the self as the collective unconscious life experience of human beings, which is the harmony and balance of various opposing forces in the human mind (Jung and Jaffé, 1963).

In recent years, Hwang Kwang-Kuo, a psychologist in Taiwan, proposed a mandala self model with universal cultural adaptability based on the mandala model of Tibetan Buddhism and Chinese Confucian culture. According to Hwang's mandala model, the so-called “self” refers to a social individual with reflexive ability, whose life world can be represented by a structural model with circles in a square (mandala). The self, as a psychological concept, lies in the center of two bidirectional arrows in the circle: one horizontal arrow points to “action” or “practice,” while the other points to “knowledge” or “wisdom.” The top of the vertical arrow points to “person” and the bottom points to “individual.” The arrangement of these concepts means that one's self is influenced by several forces from his/her life world (Hwang, 2011, 2018).

Although Hwang's mandala model of the self wants to realize the compatibility and universality of culture, the two theories it is based on cannot well explain the Chinese culture's view of the self (Wang et al., 2019). Hwang's theoretical model draws lessons from Freud's psychoanalysis and uses the iceberg theory to explain the self, dividing it into the consciousness and the unconsciousness. Furthermore, it draws lessons from the theories of Tibetan Buddhism and Indonesian Buddhism. These are unfamiliar to ordinary Han Chinese people, so it is difficult to apply them to the context of Chinese culture. Although both Chinese Buddhism and Tibetan Buddhism are related to Mahayana Buddhism, the transformation of Chinese Buddhism based on Chinese culture itself is obvious. Some scholars even believe that Chinese Buddhism is no longer Buddhism (Stone, 1999). Therefore, Hwang's self model cannot well explain the self theory in Chinese culture.

### The Taiji Model of the Self

**Figure 1:**
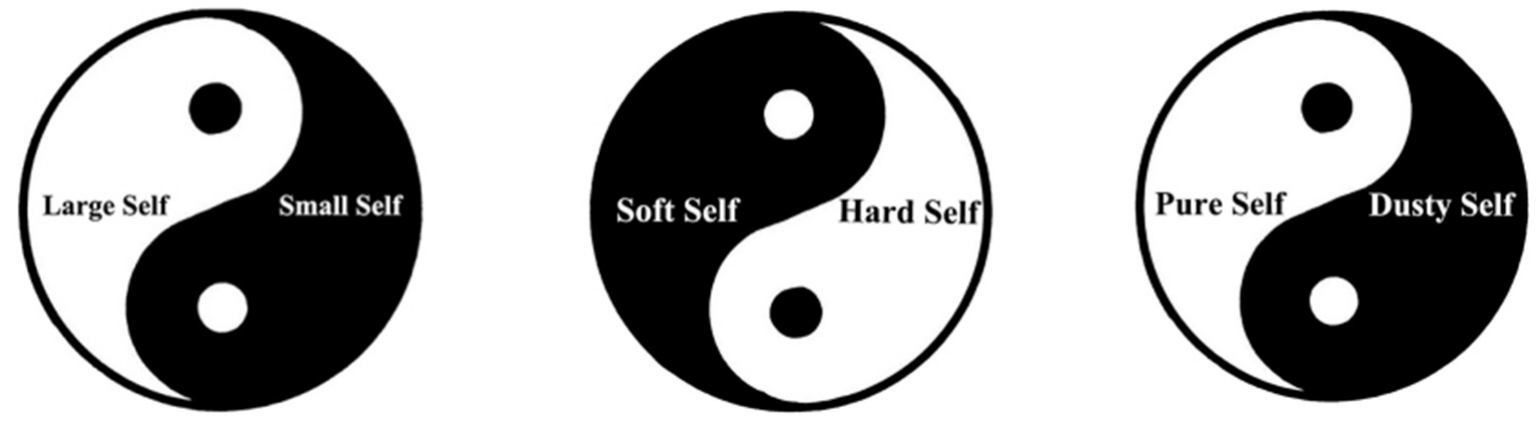
The Taiji model of self.

Inspired by the Chinese Taiji diagram, Zhen Dong Wang and others constructed the Taiji self model suitable for Chinese traditional culture. They believed that the self model of Taiji can be used to explain the self theories of Confucianism, Taoism, and Buddhism. The Taiji is composed of a circle and two parts representing Yin and Yang. In the Taiji model of Confucian self (see Figure 1), Taiji is the whole self, and Yin and Yang represent the small self and the large self, respectively. The change and transformation between Yin and Yang reflect the Confucian personality cultivation in the tradition of restraining the small self and extending the large self (Wang et al., 2019). In the Taiji model of Taoist self (see Figure 1), Yin and Yang represent the soft self and the hard self, respectively. Taoism advocates a soft self with the characteristics of softness, peace, inferiority, inaction, and lust. Therefore, the process of Taoist self-cultivation is to restrain the hard self and extend the soft self. In the Taiji model of Buddhist self (see Figure 1), the Yin part represents the dusty self (this word is more commonly expressed as “defiled self” in general Buddhist scholarship), and the Yang part represents the pure self. The goal of self-cultivation in Buddhism is to “purify the dusty self and let the pure self appear,” so as to realize the emptiness and purity of the self (Wang and Wang, 2020).

This theory has highly positive significance in explaining the Chinese self concept. However, as the author said, Chinese Confucianists, Taoists, and Buddhists have different views on the self, so the self models of Confucianists and Taoists cannot be well-applied to Buddhism. The literature he used to construct the Taiji self model of Buddhists was mainly the basic literature of Buddhism, which discusses basic theories recognized by Mahayana Buddhism, and does not reflect the characteristics of Chinese Buddhism. Therefore, it cannot be well-applied to Chinese Buddhism.

## The “One Mind, Two Aspects” Self Model

### Moral Self, Wisdom Self, Immoral Self, and Desire Self

Because Confucianism has been regarded as an official ideology by the Chinese government since the Han Dynasty, numerous psychologists have tended to discuss the Chinese self model in the context of Confucian culture (Hoshmand and Ho, 1995; Yang, 2006; Fei, 2008). However, comprehensive examination of the history of Chinese thought indicates that, since the Sui and Tang Dynasties, and particularly after Buddhism fully absorbed Confucianist and Taoist thought with the appearance of new Buddhism with Chinese characteristics, the thinking of Chinese people became increasingly influenced by Buddhism (Feng, 1933; Yang, 2003, 2016). Thus, only by understanding the construction of the self model in Chinese Buddhism can we truly understand the concept of the self in the context of Chinese culture.

The book *Awakening of Faith in the Mahāyāna*, which is widely believed to have been written by a Chinese monk, contains all of the secrets of the Chinese Buddhist self model. In contrast to Indian Buddhism and Tibetan Buddhism, this book absorbs the “substance and function (Ti Yong, 用)” theory of traditional Chinese culture and the ideological tendency of attaching importance to morality (Wawrytko, 2018; Kwon and Jeson, 2019) and proposes the psychological model of “one mind, two aspects.”

**Figure 2:**
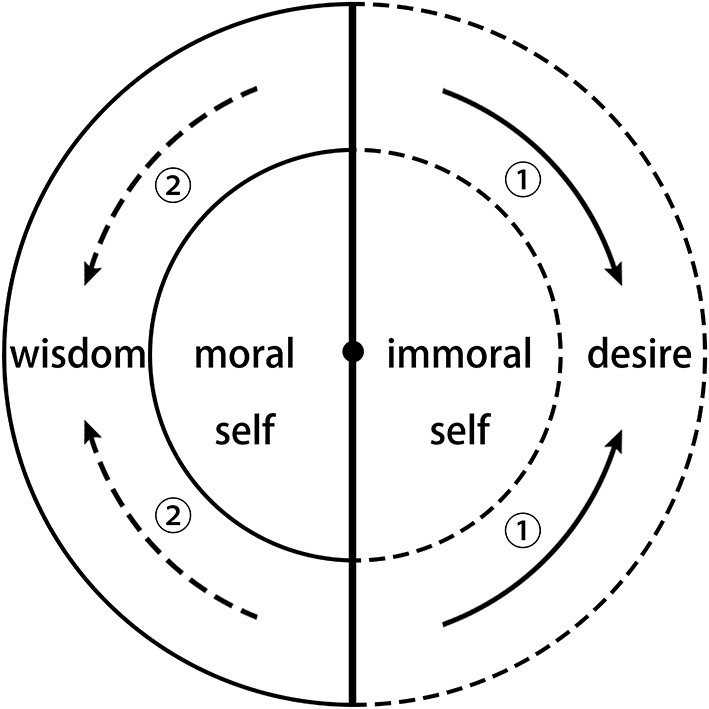
The “one mind, two aspects” model of the Chinese Buddhist self.

In Indian Buddhism, the self refers to a substance that is permanent, unchangeable, unique, independent, and autonomous. In the Chinese Buddhist self model of “one mind, two aspects,” as shown in Figure 2, the self is divided into many parts, including the moral self, the wisdom self, the immoral self, and the desire self. Each of these four are associated with the Buddha, who, as the ultimate self, represents the highest morality and wisdom, also known as “one mind.” Among these, the moral self and the wisdom self are the result of ordinary people sharing the Buddha's virtues, which is the good aspect. The immoral self and the desire self are the result of ordinary people's misunderstanding of the nature of the world and the domination by desire, which is the bad aspect. In the view of Chinese Buddhism, ordinary people tend to have both good and bad aspects, whereas the Buddha has only the good aspect. If ordinary people want to become the Buddha, they have to eliminate the bad aspects (Richard, 1907; Kwon and Jeson, 2019).

It is important to note that the good aspect, namely, the moral self and the wisdom self, is derived from the Buddha who represents the highest morality and wisdom (represented by the most central black dot in Figure 2). The moral self can lead people to conduct moral behaviors, and the wisdom self can lead people to understand the nature of the world in accordance with Buddhist philosophy. In the existing Buddhist psychological research, these two kinds of self are usually referred to as the “non-self” or the “pure self.” However, because, in Chinese Buddhism, the “non-self” represents the transcendence of the self, it usually refers to the supreme Buddha, whereas “pure self” refers to the divine existence beyond ordinary people through religious practice, such as Bodhisattva. Therefore, here we do not use these two terms, but directly use “moral self” and “wisdom self.”

The immoral self and the desire self, as the bad aspects, emerge because ordinary people do not understand the two truths of the universe and of human life. As summarized by Ch'En, the two truths are the following: First, there is an all-pervasive force called karma (業報), which operates inexorably to reward good deeds with meritorious rebirths and evil deeds with rebirth in one of the evil modes of existence (Ch'En, 1973). If people do not recognize this truth, then it is easy to produce immoral behavior in bodily action (身), speech (口), and thought (意), contributing to the immoral self (McGuire, 2013). Second, the phenomenal world is illusory, like a mirage or shadow, indicating that life is suffering and transitory, that sensual pleasures are undesirable and therefore ought to be suppressed or eradicated, and that the ideal pattern of life is withdrawal from society and family to a life of celibacy and mendicancy. If people do not realize this truth, then they will easily be dominated by desire, contributing to the desire self, and producing eight kinds of suffering: birth, old age, sickness, death, separation from loved ones, closeness to loathsome people, not getting what one wants, and the five aggregates (Ch'En, 1973).

Looking at this model from a holistic perspective, we learn that Chinese Buddhism believes that the self is fundamentally from the Buddha, and that the essence of a person is ontologically the same as the essence of the Buddha, so they can be called “one mind.” However, in real life, an ordinary person's self contains two aspects: the good aspect includes the moral self and the wisdom self, while the bad aspect includes the immoral self and the desire self. The psychological power of these two aspects is constantly changing. When a person cannot control his desires, conducts evil behaviors, and is unwilling to accept the truth of Buddhism, the desire self and the immoral self will cover up the moral self and the wisdom self according to the route shown by arrow ② in Figure 2 and will prevent the good aspect of the self from playing its roles, which will lead to the degeneration of the human. When a person acts according to the Buddhist precepts and tries to realize the truth of Buddhism, then the moral self and wisdom self will be more powerful and can restrain the bad aspect of the self according to the route shown by arrow ① in Figure 2. This can bring humanity closer to the Buddha. When the self is completely eliminated, one becomes a Buddha.

### The Self-Cultivation Theory of Chinese Buddhism

**Figure 3:**
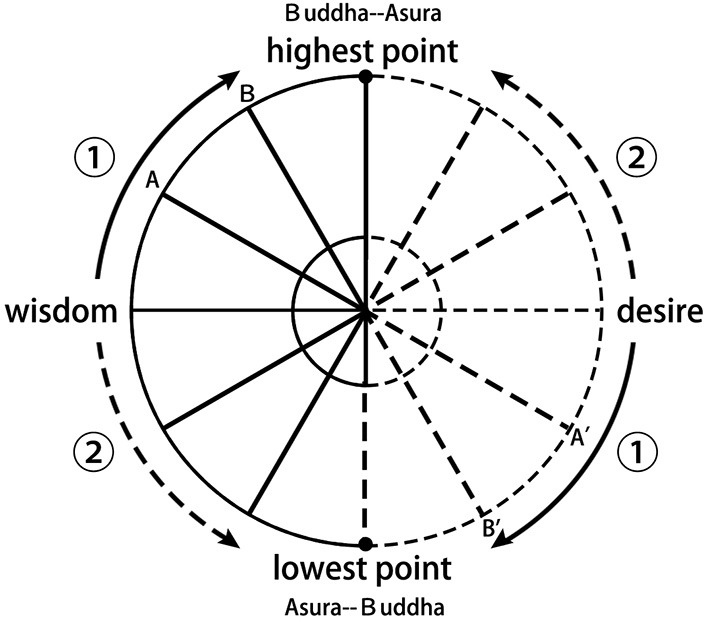
Schematic diagram of the changing of the Chinese Buddhist self.

According to the rise and fall of the power of the two aspects of the self, Chinese Buddhism makes a detailed distinction between humans and Buddha, Bodhisattva, Arahant, ghost, animal, Asura, etc. Figure 3 illustrates this distinction clearly. The horizontal straight line in the middle of the circle represents the state of the power balance of the four selves.

When the power of the moral self and the wisdom self rises to point A, the power of the immoral self and the desire self will fall to point A'. Thus, one will become an Arhat (Sanskrit), the first-level god in Buddhism. Arhats are considered to be disciples of the Buddha who have obtained enlightenment by their own efforts. They act in full accordance with the Buddhist precepts and will not violate morality (Little, 1992). They also understand the truth of Buddhism and particularly the Four Noble Truths—dukkha (suffering), samudaya (cause), magga (path), and nirodha (cessation)—and acquire the wisdom of the Buddha. Compared to Bodhisattvas, however, they have not yet made a vow to save all sentient beings, so they are also called “self-enlighteners” (Ikeda, 2001; Wang and Wang, 2020).

When the power of the moral self and the wisdom self rises to point B, the power of the immoral self and the desire self will fall to point B'. Thus, one will become a second-level god in Buddhism, i.e., Bodhisattva. Bodhisattva has all of the virtues and wisdom of the Arhat. More importantly, Bodhisattva not only can enlighten himself but also can benefit all sentient beings by helping them realize their wishes and teaching them to abide by the precepts, so that they can become Buddha in the future. However, compared with Buddha, Bodhisattva still lacks “Dzogchen” (Vorenkamp, 2004; Wang and Wang, 2020).

When the power of the moral self and the wisdom self rises to the highest point, the power of the non-moral self and the desire self will disappear, and a person becomes the highest-level god in Buddhism, i.e., Buddha. As “one mind,” Buddha is the noumenon of all sentient beings' hearts and also the highest noumenon of wisdom and morality. It is a mysterious existence that constantly produces moral behavior and knowledge of the truth (Ding, 2017).

Alternatively, as indicated by route ②, when the power of the moral self and the desire self rises along the line, the power of the moral self and the wisdom self will gradually decline, and in this process, people will become evil and painful beings, such as beasts, hungry ghosts, and Asuras.

In Chinese Buddhism, Arhats, Bodhisattvas, and Buddhas are higher than human beings in morality and wisdom, whereas beasts, hungry ghosts, and Asuras are lower than human beings in morality and wisdom. As shown in the figure, the increase and decrease between the power of moral/wisdom and immoral/desire affect the transition between the lower and higher beings. Thus, ordinary people's self-cultivation is actually to restrain their desires through basic moral principles so that they will not degenerate into low-level beings and to enhance morality and wisdom by abiding by Buddhist precepts and practicing meditation, so as to realize the transformation from human to Arhat, Bodhisattva, and even Buddha. The specific process is shown in Table 1.

**Table 1:** The self-cultivation process of Chinese Buddhism.

**Cultivationlevel**	**Types**	**Beings**	**The type of uncontrollable desire**	**Precepts**	**Meditation**
High	Non-human beings (high-level)	Buddhas		All precepts	The great meditation
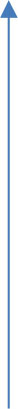		Bodhisattvas		Bodhisattva precepts^a(A)^^(B)^^(C)^	Developing wisdom, which involves mindfulness and insightful contemplation
		Arhats		Bodhisattva precepts^a(A)^^(B)^^(C)^	
	Human beings	Monks	Affinity for the Way	250 precepts for men; 348 precepts for women^b^	Focusing the mind, which calms the mind and makes it peaceful
		Lay Practitioners	Affinity for the gods	Five precepts^c^	Ethical conduct, which reduces mental proliferations relating to guilt and remorse
		Ordinary people	Love	Five precepts^c^	
Low	Non-human beings (low-level)	Beast	Craving	The 10 grave precepts^a(C)^	
		Hungry ghosts	Clinging	The 10 grave precepts^a(C)^	
		Asuras	Anger	The 10 grave precepts^a(C)^	

*^a(a)^ The three treasures: Taking refuge in the Buddha, taking refuge in the Dharma, and taking refuge in the Sangha. ^(b)^The three pure precepts: Do not create evil, practice good, and actualize good for others. ^(c)^The 10 grave precepts: Do not kill; do not steal; do not misuse sexuality; do not lie; do not cloud the mind; do not speak of others' errors and faults; do not elevate the self or blame others; do not be withholding; do not be angry; do not defile the three treasures*.

b *Including the norms of all human behaviors in life*.

c *Including refrain from killing, refrain from stealing, refrain from sexual misconduct, refrain from lying, and refrain from consuming intoxicants*.

## Conclusions

The study of self in Western psychology comes from the thinking of human mind in philosophy. At first, the philosophical concept of the ego as the cognitive subject is the logical condition to highlight the special position of man in nature. After the emergence of psychology as a science, psychologists regarded the self as a research object and began to use empirical methods to explain the self-cognition of individuals and groups and the structure of such cognition, resulting in a variety of self-concepts and models. Through the analysis of cultural psychology, we find that these theories have some limitations in explaining the self under different cultural backgrounds. It is especially difficult to explain the self model under the background of Chinese Buddhist culture, because there are great differences between them in theoretical background, theoretical basis, theoretical content, and value orientation. There are two specific manifestations of this difference. First, the self model in Western psychology always emphasizes the entity of self and strengthens the distinction between self and others, individual and external environment, and human and nature. This is essentially different from the Chinese Buddhist culture, which regards the self as an illusory existence and emphasizes the concept of eliminating the individual self. The second is that both the object self and the collective self affirm the legitimacy of the individual self, and its ultimate purpose is to better stimulate the potential of the self and achieve self-satisfaction and happiness through self-knowledge. In the context of Chinese Buddhist culture, the self-theory is to transform the self into non-self through discipline and meditation, so as to achieve the improvement of moral quality and spiritual realm.

Compared to existing models of non-self based on Eastern culture, the “one mind, two aspects” self model in *Awakening of Faith in the Mahāyāna* can properly express the self structure of Chinese in the context of Chinese Buddhism. “One mind” is the ultimate self represented by Buddha. The “two aspects” correspond to the good aspect (including moral self and wisdom self) and the bad aspect (including immoral self and desire self). The change and transformation between the two aspects reflect the self-cultivation of Buddhism in the tradition of expanding the moral self and the wisdom self and eliminating the immoral self and the desire self.

On the whole, the one mind–two aspects self model denies the subject–object dichotomy of self construction mode in the context of Western culture. In this new model, not only people's perception of self has changed, but the relationship between human and the external world, especially human and nature, is also fundamentally different from that of the West. In the Western self model, the self is an entity, so it is a legitimate choice to maintain the self by sacrificing the environment. However, Chinese Buddhism believes that only things that are eternal and unchanging are entities that are worthy of being pursued. Besides the highest morality and wisdom represented by the Buddha, there is no unchanging entity in this world. Self and the nature on which one lives are full of changes; both are considered to be non-entity existence and therefore should not be maintained, nor are they worthy of being pursued. Under this theoretical background, the one mind–two aspects self model emphasizes that individuals and society, human, and nature are regarded as unreal illusions that are full of changes, easy to be fleeting; the focus of individual attention should shift from the outer world to the inner world; in real life, people should pay attention to moral improvement and spiritual purification, rather than using nature to satisfy one's own desires. This mode of discussion helps us deepen our understanding of self-theories in different cultural contexts, thereby providing new ideas for us to think about the relationship between ourselves and the external environment.

## Author Contributions

The author confirms being the sole contributor of this work and approved it for publication.

## Conflict of Interest

The author declares that the research was conducted in the absence of any commercial or financial relationships that could be construed as a potential conflict of interest.

## References

[B1] Allport F. H. (1962). A structuronomic conception of behavior: individual and collective. I. Structural theory and the master problem of social psychology. J. Abnorm. Soc. Psychol. 64, 3–30. 10.1037/h0043563 13860640

[B2] Brewer M. B. (1988). A dual process model of impression formation, in Advances in Social Cognition: A Dual Process Model of Impression Formation, Vol. 1, eds Srull T. K. Wyer R. S. (Hillsdale: Erlbaum), 1–36.

[B3] Brewer M. B. Gardner W. (1996). Who is this we? Levels of collective identity and self-representations. J. Pers. Soc. Psychol. 71, 83–93. 10.1037/0022-3514.71.1.83

[B4] Cartwright D. (1979). Contemporary social psychology in historical perspective. Soc. Psychol. Q. 42, 82–93. 10.2307/3033880

[B5] Ch'En K. K. S. (1973). Chinese Tranformation of Buddhism. Princeton, NJ: Princeton University Press.

[B6] Cooley C. H. (1902). Human Nature and the Social Order. New York, NY: Charles Scribner's Sons.

[B7] Ding F. B. (2017). The Dictionary of Buddhism. Nanjing: Jiangsu People's Publishing House.

[B8] Fei H. (2008). From the Soil: The Foundations of Chinese Society. Beijing: People's Publishing House.

[B9] Feng Y. L. (1933). History of Chinese Philosophy. Beijing: The Commercial Press.

[B10] Fitts W. H. (1965). Tennessee Self Concept Scale. Los Angeles: Western Psychological Services.

[B11] Fox K. R. Corbin C. B. (1989). The psychology self-perception profile: development and preliminary validation. J. Sport Exer. Psychol. 11, 408–430. 10.1123/jsep.11.4.40817624480

[B12] Gergen K. (2001). Psychological science in a postmodern world. Am. Psychol. 56, 803–813. 10.1037/0003-066X.56.10.803 11675987

[B13] Giorgi A. (2005). Remaining challenges for humanistic psychology. J. Hum. Psychol. 45, 204–216. 10.1177/0022167804274361

[B14] Graumann C. F. (1986). The individualization of the social and the desocialization of the individual: Floyd H. Allport's contribution to social psychology, in Changing Conceptions of Crowd Mind and Behavior, eds Graumann C. F. Moscovici S. (New York, NY: Springer-Verlag), 97–116. 10.1007/978-1-4612-4858-3_7

[B15] Hogg M. Williams K. (2000). From I to We: social identity and the collective self. Group Dynamics 4:81. 10.1037/1089-2699.4.1.81

[B16] Hoshmand L. T. Ho D. Y. F. (1995). Moral dimensions of selfhood: chinese traditions and cultural change. World Psychol. 1, 47–69.

[B17] Hwang K. K. (2011). The mandala model of self. Psychol. Stud. 56:329. 10.1007/s12646-011-0110-1

[B18] Hwang K. K. (2018). A psychodynamic model of self-nature. Counsel. Psychol. Quart. 32, 1–22. 10.1080/09515070.2018.1553147

[B19] Ikeda D. (2001). My View of Buddhism (B. T. L. -Q., Trans.). Chengdu: Sichuan People's Publishing House.

[B20] Jung C. Jaffé A. (1963). Memories, Dreams, Reflections. New York, NY: Pantheon Books.

[B21] Kwon S. h. Jeson W. (2019). On the origin and conceptual development of ‘Essence-Function' (ti-yong). Religions 10:272. 10.3390/rel10040272

[B22] LeBon G. (1908). The Crowd: A Study of the Popular Mind. London: Unwin. 10.1037/10878-000

[B23] Little S. (1992). The arhats in China and Tibet. Artibus Asiae 52, 255–281. 10.2307/3249891

[B24] Markus H. R. Kitayama S. (1991). Culture and the self: implications for cognition, emotion, and motivation. Psychol. Rev. 98, 223–253. 10.1037/0033-295X.98.2.224

[B25] Marsh H. W. (1992). Content specificity of relations betweenacademic achievement and academic self-concep. J. Educ. Psychol. 84, 35–42. 10.1037/0022-0663.84.1.35

[B26] Marsh H. W. Redmayne R. S. (1994). A multidimensional physical self-concept and its relation to multiple components of physical fitness. J. Sport Exer. Psychol. 16, 45–55. 10.1123/jsep.16.1.43

[B27] McDougall W. (1921). The Group Mind. London: Cambridge University Press.

[B28] McGuire B. F. (2013). Divining karma in Chinese Buddhism. Religion Compass 7, 413–422. 10.1111/rec3.12068

[B29] Mead G. H. (1925). The genesis of the self and social control. Int. J. Ethics 35, 251–277. 10.1086/intejethi.35.3.2377274

[B30] Mead G. H. (1934). Mind, Self and Society, Vol. 111. Chicago: University of Chicago Press.

[B31] Moscovici S. (1984). The Phenomenon of Social Representations Social Representations. Cambridge: Cambridge University Press.

[B32] Neisser U. (1988). Five kinds of self-knowledge. Philos. Psychol. 1, 35–59. 10.1080/09515088808572924

[B33] Oosterwegel J. H. Oosterwegel A. Oppenheimer L. Oppenheimer L. J. T. (1993). The Self-System: Developmental Changes Between and Within Self-concepts. Hillsdale: L Erlbaum.

[B34] Richard T. (1907). The Awakening of Faith in the Mahayana Doctrine: The New Buddhism. Shanghai: Christian Literature Society.

[B35] Rogers C. (1951). Client-Centered Therapy: Its Current Practice, Implications and Theory. London: Constable.

[B36] Shear J. Gallagher S. (2013). Models of the Self. Exeter: Imprint Academic.

[B37] Shiah Y.-J. (2016). From self to nonself: the nonself theory. Front. Psychol. 7:124. 10.3389/fpsyg.2016.00124 26869984 PMC4740732

[B38] Sonstroem R. J. Morgan W. P. (1989). Exercise and self-esteem rationale and model. Med. Sci. Sports Exerc. 21:333. 10.1249/00005768-198906000-00018 2659918

[B39] Stone J. (1999). Some Reflections on Critical Buddhism. [Pruning the Bodhi Tree: The Storm over Critical Buddhism, Jamie Hubbard, Paul L. Swanson]. Jpn. J. Relig. Stud. 26, 159–188.

[B40] Tajfel H. (1972). La categorisation sociale, in Introduction a la psychologie sociale, Vol. 1, ed S. Moscovici (Paris: Larousse), 272–302.

[B41] Tajfel H. (1974). Intergroup Behaviour, Social Comparison and Social Change. Ann Arbor: University of Michigan.

[B42] Talhelm T. Zhang X. Oishi S. Shimin C. Duan D. Lan X. (2014). Large-scale psychological differences within China explained by rice versus wheat agriculture. Science 344, 603–608. 10.1126/science.1246850 24812395

[B43] Tarde G. (1901). L'opinion et la foule. Paris: Libraire Felix Alcan.

[B44] Triandis H. C. (1989). The self and social behavior in differing cultural contexts. Psychol. Rev. 96, 506–520. 10.1037/0033-295X.96.3.506

[B45] Vorenkamp D. (2004). Evil, The Bodhisattva Doctrine, and Faith in Chinese Buddhism: examining Fa Zang's three tests. J. Chin. Philos. 31, 253–269. 10.1111/j.1540-6253.2004.00153.x

[B46] Walker L. J. (2004). Progress and prospects in the psychology of moral development. Merrill Palmer Q. 50, 546–557. 10.1353/mpq.2004.0038

[B47] Wang F. Y. Wang Z. D. Wang R. J. (2019). The Taiji model of self. Front. Psychol. 10:1443. 10.3389/fpsyg.2019.01443: 31293484 PMC6598445

[B48] Wang Z. D. Wang F. Y. (2020). The Taiji model of self II: developing self models and self-cultivation theories based on the Chinese cultural traditions of Taoism and Buddhism. Front. Psychol. 11:540074. 10.3389/fpsyg.2020.540074 33178061 PMC7591803

[B49] Wawrytko S. A. (2018). The Sinification of Buddhist Philosophy: the Cases of Zhi Dun and The awakening of faith in the Mahāyāna (Dasheng Qixin Lun),in Dao Companion to Chinese Buddhist Philosophy. Dao Companions to Chinese Philosophy, Vol. 9, eds Wang Y. Wawrytko S. A. (Dordrecht: Springer), 29–44. 10.1007/978-90-481-2939-3_2

[B50] William J. (1890). The Principles of Psychology. New York, NY: Henry Holt and Company.

[B51] William J. (1892). Psychology: The Briefer Course. New York, NY: Henry Holt.

[B52] Wundt W. (1916). Elements of Folk Psychology:Outlines of a Psychological History of the Development of Mankind. London: Allen and Unwin. 10.1037/13042-000

[B53] Yang C.-F. (2006). The Chinese conception of the self towards a person-making perspective, in Indigenous and Cultural Psychology: Understanding People in Context, eds Yang K.-S. Kim U. Hwang K. K. (New York: NY: Springer), 327–356.

[B54] Yang K. S. Lu L. (2009). Chinese Self: Analysis With Psychology. Chongqing: Chongqing University Press.

[B55] Yang W. Z. (2003). The substance of the nature of mind and the substance of Taoist nature: the influence of Buddhist theory about the nature of mind of China upon Taoist Theory about the nature of mind. Stud. World Relig. 2, 63–72. 10.3969/j.issn.1000-4289.2003.02.008

[B56] Yang W. Z. (2016). On the influence of Buddhist mind ontology on confucian theories during song and ming dynasties. J. Jiangsu Administr. Ins. 88, 5–12. 10.3969/j.issn.1009-8860.2016.04.001

[B57] Zhan Q. Yue G. a. (2002). A Hundred and More Years of the Studying of Ego:a Historical Retrospect and its Prospective Development. Nankai Journal Philosophy and Social Science Edition, 5, 27–33.

[B58] Zhu Y. Ng X. H. (2017). Looking for the Chinese Self. Beijing: Beijing Normal University Press.

